# Synthesis, crystal structure and Hirshfeld surface analysis of 5-methyl-2-[(1,3-thia­zol-2-yl)sulfan­yl]-1,3,4-thia­diazole

**DOI:** 10.1107/S2056989025004980

**Published:** 2025-06-06

**Authors:** Ekaterina Kinshakova, Batirbay Torambetov, Manoj K Bharty, Aziz Atashov, Abdusamat Rasulov, Shakhnoza Kadirova, Rajesh G Gonnade

**Affiliations:** a National University of Uzbekistan named after Mirzo Ulugbek 4 University St Tashkent 100174 Uzbekistan; b Physical and Material Chemistry Division CSIR-National Chemical Laboratory,Pune 411008 India; c Department of Chemistry, Banaras Hindu University, Varanasi 221 005, India; d Karakalpak State University, 1 Ch. Abdirov St. Nukus, 230112, Uzbekistan; e Termez University of Economics and Service, 41B Farovon St., Termiz, 190111, Uzbekistan; f Academy of Scientific and Innovative Research (AcSIR) Sector 19 Kamla Nehru Nagar Ghaziabad Uttar Pradesh 201002 India; Indian Institute of Science Education and Research Bhopal, India

**Keywords:** 1,3,4-thia­diazole, 1,3-thia­zole, C—H⋯N inter­action, crystal structure, Hirshfeld surface analysis

## Abstract

The title compound, C_6_H_5_N_3_S_3_, consists of two biologically relevant heterocyclic units, suggesting potential biological activity and possible use as a ligand in metal complexation. The compound crystallizes in the monoclinic space group *P*2_1_/*c* and features non-classical inter­molecular C—H⋯N hydrogen bonds, along with π–π stacking inter­actions that contribute to the crystal cohesion. Hirshfeld surface analysis highlights significant inter­molecular inter­actions including, among others, N⋯H/H⋯N, S⋯H/H⋯S, and S⋯C/C⋯S contacts.

## Abstract 2

The title compound contains two biologically active heterocyclic rings, 1,3,4-thia­diazole and 1,3-thia­zole, connected *via* a sulfur atom. The packing is consolidated by non-classical inter­molecular C—H⋯N hydrogen bonds and *π*–*π* stacking inter­actions.

## 1. Chemical context

Derivatives combining 1,3,4-thia­diazole and 1,3-thia­zole moieties offer significant potential in medicinal chemistry due to their enhanced biological activity, pharmacokinetic profiles and structural versatility. This class of compounds is being actively explored in various therapeutic areas, including their use as anti­microbial (Booq *et al.*, 2021 ▸; Hussain *et al.*, 2022 ▸), anti­cancer (Shaikh *et al.*, 2024 ▸; Altıntop *et al.*, 2017 ▸; Dawood *et al.*, 2013 ▸), anti-inflammatory (Arshad *et al.*, 2022 ▸) and neuroprotective agents. With ongoing research into their SAR, bioavailability, and environmental impact, these derivatives are promising candidates for the next generation of drug development.

The structural fusion of 1,3,4-thia­diazole and 1,3-thia­zole is expected to have synergistic biological effects due to their different modes of action. Thia­diazo­les are often involved in enzyme inhibition and inter­action with metal ions, while thia­zoles enhance inter­actions with biological targets such as nucleic acids or proteins.

Herein, we report the synthesis and crystal structure of a new heterocyclic compound with combination of 1,3,4-thia­diazole and 1,3-thia­zole fragments. This 2-thia­zole-substituted derivative can act as a chelating ligand.
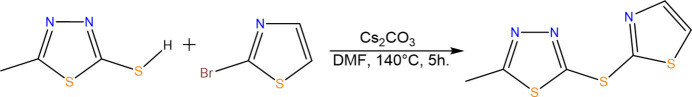


## 2. Structural commentary

**Figure 1:**
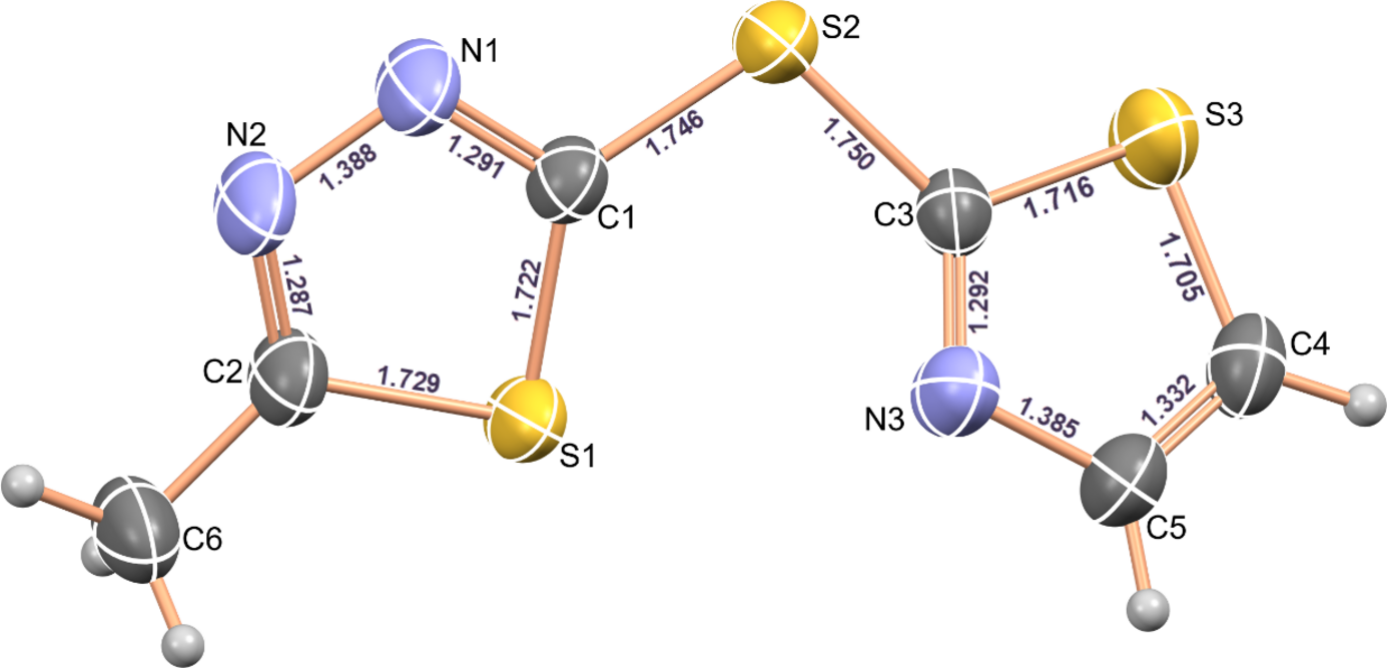
A view of the mol­ecular structure of 5-methyl-2-[(1,3-thia­zol-2-yl)sulfan­yl]-1,3,4-thia­diazole, showing the atom labeling and bond lengths. Displacement ellipsoids are drawn at the 50% probability level.

The title compound (Fig. 1 ▸) crystallizes in the monoclinic system, space group *P*2_1_/*c*. The mol­ecular structure of the compound is shown in Fig. 1 ▸. The geometric parameters of the thia­diazole and thia­zole rings are close to standard values and the values reported for related structures. (Renier *et al.*, 2023 ▸; Luqman *et al.*, 2016 ▸; Burnett *et al.*, 2015 ▸; Dani *et al.*, 2014 ▸; Weidner *et al.*, 2008 ▸; Jumal *et al.*, 2006 ▸; Kennedy *et al.*, 2004 ▸; Hipler *et al.*, 2003 ▸). The N—N and endocyclic C—S bonds are shorter than classical single bonds (1.4 and 1.81 Å), indicating partial double-bond character. At the same time, the C=N bonds are somewhat longer (∼0.02 Å) than the corresponding double bond, as a result of conjugation within the ring systems. These facts confirm the aromaticity of both rings. The exocyclic C—S bond is shortened since it includes carbon atoms with *sp*^2^ hybridization. Deviation of the bond angles from 120° in the 1,3,4-thia­diazole and 1,3-thia­zole rings is a common feature in five-membered rings (Bharty *et al.*, 2012 ▸). The C—S—C bond angles in the 1,3,4-thia­diazole and 1,3-thia­zole rings of the title compound are 86.62 (8) and 89.25 (9)°, respectively, and the C1—S2—C3 bond angle outside the ring is 103.82 (8)°. The thia­diazole and thia­zole rings do not lie in the same plane, subtending a dihedral angle of 32.61 (10)°. No intra­molecular hydrogen bonds are observed.

## 3. Supra­molecular features and energy framework calculations

**Figure 2:**
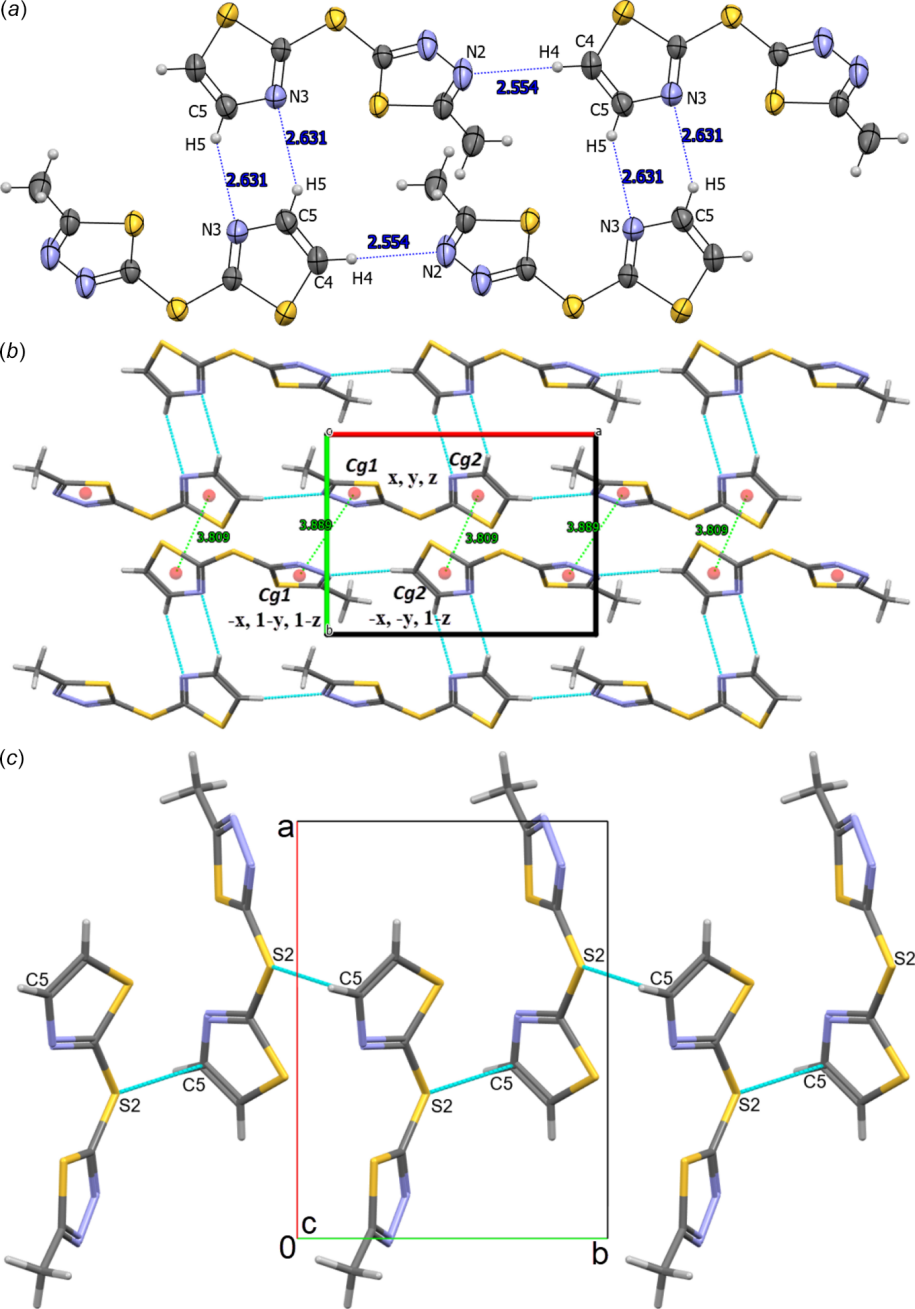
(*a*) Overview of inter­molecular C4—H4⋯N2 and C5—H5⋯N3 hydrogen bonds (shown in blue), (*b*) a view of the *π*–*π* stacking inter­actions (hydrogen bonds are shown in blue and *π*–*π* stacking inter­actions are shown in green). and (*c*) Highlight of S2⋯C5 chalcogen inter­actions (dashed lines) along the *b*-axis direction, with relevant atoms labeled.

The crystal packing is consolidated by C5—H5⋯N3^ii^ hydrogen bonds [symmetry code: (ii) *x* + 1, *y*, *z* + 1], forming a six-membered 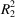 (6) ring motif (Grabowski, 2020 ▸; Etter *et al.*, 1990 ▸). Along the *a-*axis direction, cohesion of the crystal packing is achieved by C4—H4⋯N2^i^ hydrogen bonds [symmetry code: (i) *x* + 1, *y*, *z*] between the methine group of the 1,3-thia­zole ring and the nitro­gen atom of the 1,3,4-thia­diazole ring of a nearby mol­ecule. The geometrical parameters of inter­molecular hydrogen bonds are shown in Table 1 ▸ and Fig. 2 ▸*a*.

In the supra­molecular structure of the compound, weak *π*–*π*-stacking inter­actions are found (Fig. 2 ▸*b*) between thia­diazole rings (symmetry operation −*x*, 1 − *y*, 1 − *z*) with an intra­centroid distance of 3.889 (9) Å and between thia­zole rings (symmetry operation −*x*, −*y*, 1 − *z*) with a centroid-to-centroid distance of 3.809 (9) Å. Similarly, the structure also exhibits inter­molecular chalcogen bond between C5 of the thia­zole ring and the bridging S2 atom [C5⋯S2(1 − *x*, −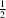 + *y*, 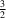 − *z*) = 3.491 (2) Å] (Fig. 2 ▸*c*).

The inter­action energies of the hydrogen-bond system were calculated within the mol­ecules using the B3LYP method (B3LYP/6-31G (d, p) in *CrystalExplorer 21.5* (Spackman *et al.*, 2021 ▸). The total energy (*E*_tot_) is the sum of Coulombic (*E*_ele_), polar (*E*_pol_), dispersion (*E*_dis_) and repulsive (*E*_rep_) contributions. The four energy components were scaled in the total energy: *E***_tot_** = 1.057*E*_ele_ + 0.74*E*_pol_ + 0.871*E*_dis_ + 0.618*E*_rep_. The inter­action energies were investigated for a 3.8 Å cluster around the reference mol­ecule. The results give a total inter­action energy of −141 kJ mol^−1^ involving electrostatic (−74.3 kJ mol^−1^), polarization (−12.2 kJ mol^−1^), dispersion (−146.9 kJ mol^−1^) and repulsion (125 kJ mol^−1^) components.

## 4. Hirshfeld surface analysis

**Figure 3:**
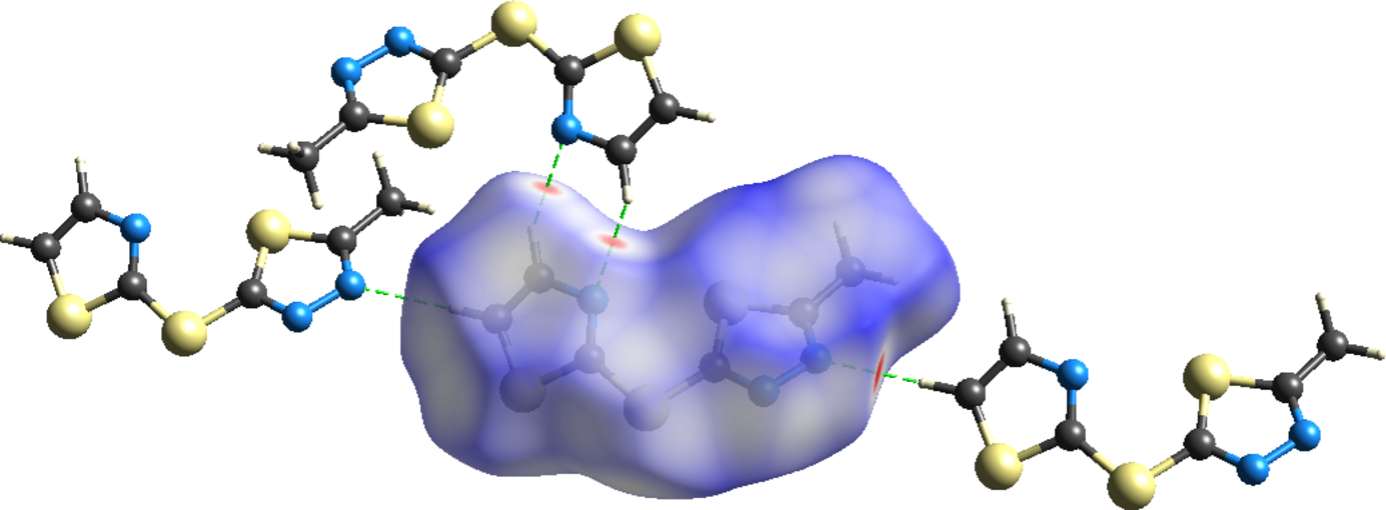
View of the three-dimensional Hirshfeld surface of the mol­ecule plotted over *d*_norm_.

To further investigate the inter­mol­ecular inter­actions present in the title compound, a Hirshfeld surface analysis was performed, and the two-dimensional (2D) fingerprint plots were generated with *CrystalExplorer17* (Spackman *et al.*, 2021 ▸). Fig. 3 ▸ shows the three-dimensional (3D) Hirshfeld surface of the complex plotted over *d*_norm_ (normalized contact distance). The hydrogen-bond inter­actions given in Table 1 ▸ play a key role in the mol­ecular packing of the complex.

**Figure 4:**
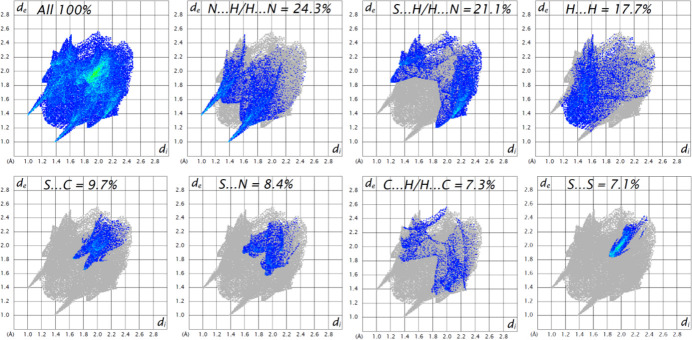
The full two-dimensional fingerprint plot for the title compound, showing all inter­actions, and those delineated into separate inter­actions with the percentage contributions of various inter­atomic contacts occurring in the crystal.

The overall 2D fingerprint plot and those divided into inter­atomic inter­actions are shown in Fig. 4 ▸. The Hirshfeld surface analysis shows that 24.3% of the inter­molecular inter­actions are from N⋯H/H⋯N contacts, 21.1% from S⋯H/H⋯S contacts, 17.7% from H⋯H contacts and 9.7% are from S⋯C/C⋯S contacts, while other contributions are from C⋯H/H⋯C, S⋯C/C⋯S and S⋯N/N⋯S contacts (Fig. 4 ▸).

## 5. Database survey

A survey of the Cambridge Structural Database performed using ConQuest software (CSD, Version 5.46, last updated November 2024; Groom *et al.*, 2016 ▸) revealed that 122 crystal structures have been reported for the 2-methyl-1,3,4-thia­diazole-5-thiol fragment; among them, 73 structures are related to organometallic compounds. There are mostly organic thiol-substituted compounds reported, because of the good reactivity of the thiol group. In addition, there are three organic structures based on the 2-methyl-1,3,4-thia­diazole-5-thiol fragment (CILHAI, Dani *et al.*, 2013 ▸; GEXWOY, Zhao *et al.*, 2010 ▸; XICMOO, Cabral *et al.*, 2018 ▸), which can bind in a bidentate manner with metal atoms to form six-membered rings. Similar to C_6_H_5_N_3_S_3_, chalcogen-bonding inter­actions were observed in both structures. In CILHAI, S—N chalcogen inter­actions occur where both nitro­gen atoms of the thia­diazole ring inter­act with the bridging sulfur atom and the sulfur atom of an adjacent thia­diazole ring. In XICMOO, a chalcogen inter­action is present between a sulfur atom and a carbon atom of a neighboring benzene ring.

## 6. Synthesis and crystallization

A solution of 5-methyl-1,3,4-thia­diazole-2-thiol (0.01 mol) and 2-bromo­thia­zole (0.01 mol) in DMF (10 ml) in presence of cesium carbonate was stirred for 5 h at 413 K. DMF was distilled off with a rotary evaporator. The resulting brown concentrate was dissolved in DCM/MeOH and separated by flash column chromatography. The synthesized amorphous product 2-methyl-5-(1,3-thia­zol-2-ylsulfan­yl)-1,3,4-thia­diazole was light yellow in color (m.p. 327 K). Further recrystallization gave crystals suitable for X-ray diffraction (yield: 60%).

## 7. Refinement

Crystal data, data collection and structure refinement details are summarized in Table 2 ▸. H atoms were positioned geometrically (C—H = 0.93–0.96 Å) and refined as riding with *U*_iso_(H) = 1.2*U*_eq_(C) or 1.5*U*_eq_(C-meth­yl).

## Supplementary Material

Crystal structure: contains datablock(s) I. DOI: 10.1107/S2056989025004980/dx2067sup1.cif

Structure factors: contains datablock(s) I. DOI: 10.1107/S2056989025004980/dx2067Isup3.hkl

CCDC reference: 2455808

Additional supporting information:  crystallographic information; 3D view; checkCIF report

## Figures and Tables

**Table 1:** Hydrogen-bond geometry (Å, °)

*D*—H⋯*A*	*D*—H	H⋯*A*	*D*⋯*A*	*D*—H⋯*A*
C4—H4⋯N2^i^	0.93	2.55	3.472 (2)	169
C5—H5⋯N3^ii^	0.93	2.63	3.392 (3)	139

Symmetry codes: (i) 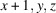; (ii) 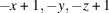.

**Table 2:** Experimental details

Crystal data	
Chemical formula	C_6_H_5_N_3_S_3_
*M*_r_	215.31
Crystal system, space group	Monoclinic, *P*2_1_/*c*
Temperature (K)	296
*a*, *b*, *c* (Å)	10.6463 (2), 7.7151 (2), 11.1774 (3)
β (°)	103.272 (1)
*V* (Å^3^)	893.56 (4)
*Z*	4
Radiation type	Mo *K*α
μ (mm^−1^)	0.77
Crystal size (mm)	0.13 × 0.1 × 0.06

Data collection	
Diffractometer	Bruker D8 VENTURE Kappa Duo PHOTON II CPAD
Absorption correction	Multi-scan (*SADABS*; Krause *et al.*, 2015 ▸)
No. of measured, independent and observed [*I* > 2σ(*I*)] reflections	18027, 2296, 1988
*R*_int_	0.052
(sin θ/λ)_max_ (Å^−1^)	0.677

Refinement	
*R*[*F*^2^ > 2σ(*F*^2^)], *wR*(*F*^2^), *S*	0.034, 0.088, 1.05
No. of reflections	2296
No. of parameters	110
H-atom treatment	H-atom parameters constrained
Δρ_max_, Δρ_min_ (e Å^−3^)	0.50, −0.48

Computer programs: *APEX2* and *SAINT* (Bruker, 2016 ▸), *SHELXT2014/5* (Sheldrick, 2015*a* ▸), *SHELXL2016/6* (Sheldrick, 2015*b* ▸) and *OLEX2* (Dolomanov *et al.*, 2009 ▸).

## supplementary crystallographic information

### 5-Methyl-2-[(1,3-thiazol-2-yl)sulfanyl]-1,3,4-thiadiazole Crystal data

**Table:**

C_6_H_5_N_3_S_3_	*F*(000) = 440
*M**_r_* = 215.31	*D*_x_ = 1.600 Mg m^−^^3^
Monoclinic, *P*2_1_/*c*	Mo *K*α radiation, λ = 0.71073 Å
*a* = 10.6463 (2) Å	Cell parameters from 8027 reflections
*b* = 7.7151 (2) Å	θ = 3.5–28.7°
*c* = 11.1774 (3) Å	µ = 0.77 mm^−^^1^
β = 103.272 (1)°	*T* = 296 K
*V* = 893.56 (4) Å^3^	Block, colourless
*Z* = 4	0.13 × 0.1 × 0.06 mm

### 5-Methyl-2-[(1,3-thiazol-2-yl)sulfanyl]-1,3,4-thiadiazole Data collection

**Table:**

Bruker D8 VENTURE Kappa Duo PHOTON II CPAD diffractometer	1988 reflections with *I* > 2σ(*I*)
φ and ω scans	*R*_int_ = 0.052
Absorption correction: multi-scan (SADABS; Krause *et al.*, 2015)	θ_max_ = 28.7°, θ_min_ = 3.6°
	*h* = −13→14
18027 measured reflections	*k* = −10→10
2296 independent reflections	*l* = −15→15

### 5-Methyl-2-[(1,3-thiazol-2-yl)sulfanyl]-1,3,4-thiadiazole Refinement

**Table:**

Refinement on *F*^2^	Primary atom site location: structure-invariant direct methods
Least-squares matrix: full	Hydrogen site location: inferred from neighbouring sites
*R*[*F*^2^ > 2σ(*F*^2^)] = 0.034	H-atom parameters constrained
*wR*(*F*^2^) = 0.088	*w* = 1/[σ^2^(*F*_o_^2^) + (0.0307*P*)^2^ + 0.4384*P*] where *P* = (*F*_o_^2^ + 2*F*_c_^2^)/3
*S* = 1.05	(Δ/σ)_max_ = 0.001
2296 reflections	Δρ_max_ = 0.50 e Å^−^^3^
110 parameters	Δρ_min_ = −0.48 e Å^−^^3^
0 restraints	

### 5-Methyl-2-[(1,3-thiazol-2-yl)sulfanyl]-1,3,4-thiadiazole Special details

**Table:**

Geometry. All esds (except the esd in the dihedral angle between two l.s. planes) are estimated using the full covariance matrix. The cell esds are taken into account individually in the estimation of esds in distances, angles and torsion angles; correlations between esds in cell parameters are only used when they are defined by crystal symmetry. An approximate (isotropic) treatment of cell esds is used for estimating esds involving l.s. planes.

### 5-Methyl-2-[(1,3-thiazol-2-yl)sulfanyl]-1,3,4-thiadiazole Fractional atomic coordinates and isotropic or equivalent isotropic displacement parameters (Å^2^)

**Table:**

	*x*	*y*	*z*	*U*_iso_*/*U*_eq_	
S1	0.18439 (4)	0.24766 (7)	0.46938 (4)	0.04704 (14)	
S2	0.34737 (4)	0.41561 (7)	0.71120 (4)	0.04642 (14)	
S3	0.61254 (5)	0.45960 (7)	0.67293 (6)	0.05644 (16)	
N1	0.10297 (15)	0.3506 (3)	0.65446 (15)	0.0558 (4)	
N2	−0.00530 (15)	0.2958 (3)	0.56825 (16)	0.0591 (5)	
N3	0.46784 (15)	0.2117 (2)	0.57237 (16)	0.0497 (4)	
C1	0.20708 (15)	0.3340 (2)	0.61488 (15)	0.0384 (3)	
C2	0.02170 (17)	0.2417 (3)	0.46812 (17)	0.0449 (4)	
C3	0.47049 (15)	0.3452 (2)	0.64267 (14)	0.0358 (3)	
C4	0.67326 (17)	0.3165 (3)	0.58452 (17)	0.0460 (4)	
H4	0.756109	0.320593	0.570753	0.055*	
C5	0.58447 (17)	0.1979 (3)	0.53809 (18)	0.0479 (4)	
H5	0.599905	0.110958	0.485673	0.058*	
C6	−0.07813 (19)	0.1783 (4)	0.3609 (2)	0.0606 (6)	
H6A	−0.089445	0.261709	0.295572	0.091*	
H6B	−0.158370	0.162416	0.384862	0.091*	
H6C	−0.050886	0.069815	0.333151	0.091*	

### 5-Methyl-2-[(1,3-thiazol-2-yl)sulfanyl]-1,3,4-thiadiazole Atomic displacement parameters (Å^2^)

**Table:**

	*U*^11^	*U*^22^	*U*^33^	*U*^12^	*U*^13^	*U*^23^
S1	0.0320 (2)	0.0677 (3)	0.0452 (2)	−0.00771 (18)	0.01673 (17)	−0.0140 (2)
S2	0.0357 (2)	0.0601 (3)	0.0451 (2)	−0.00303 (18)	0.01261 (17)	−0.0143 (2)
S3	0.0371 (2)	0.0589 (3)	0.0750 (4)	−0.0130 (2)	0.0165 (2)	−0.0186 (3)
N1	0.0346 (7)	0.0911 (13)	0.0452 (8)	−0.0027 (8)	0.0164 (6)	−0.0116 (9)
N2	0.0312 (7)	0.0991 (14)	0.0502 (9)	−0.0052 (8)	0.0160 (7)	−0.0104 (9)
N3	0.0372 (7)	0.0557 (9)	0.0591 (9)	−0.0067 (7)	0.0175 (7)	−0.0144 (8)
C1	0.0336 (7)	0.0455 (9)	0.0382 (8)	0.0005 (6)	0.0128 (6)	0.0004 (7)
C2	0.0313 (8)	0.0600 (11)	0.0456 (9)	−0.0036 (7)	0.0131 (7)	−0.0005 (8)
C3	0.0292 (7)	0.0421 (8)	0.0354 (7)	0.0000 (6)	0.0058 (6)	0.0023 (6)
C4	0.0319 (8)	0.0571 (11)	0.0507 (10)	0.0024 (7)	0.0131 (7)	0.0058 (8)
C5	0.0387 (9)	0.0568 (11)	0.0512 (10)	0.0035 (8)	0.0163 (8)	−0.0054 (8)
C6	0.0377 (9)	0.0926 (17)	0.0509 (11)	−0.0108 (10)	0.0087 (8)	−0.0101 (11)

### 5-Methyl-2-[(1,3-thiazol-2-yl)sulfanyl]-1,3,4-thiadiazole Geometric parameters (Å, º)

**Table:**

S1—C1	1.7222 (17)	N3—C3	1.292 (2)
S1—C2	1.7295 (17)	N3—C5	1.385 (2)
S2—C1	1.7460 (17)	C2—C6	1.490 (3)
S2—C3	1.7496 (16)	C4—H4	0.9300
S3—C3	1.7162 (16)	C4—C5	1.332 (3)
S3—C4	1.705 (2)	C5—H5	0.9300
N1—N2	1.388 (2)	C6—H6A	0.9600
N1—C1	1.291 (2)	C6—H6B	0.9600
N2—C2	1.287 (2)	C6—H6C	0.9600
			
C1—S1—C2	86.62 (8)	N3—C3—S3	115.16 (12)
C1—S2—C3	103.82 (8)	S3—C4—H4	125.0
C4—S3—C3	89.25 (9)	C5—C4—S3	109.93 (13)
C1—N1—N2	111.92 (15)	C5—C4—H4	125.0
C2—N2—N1	112.76 (15)	N3—C5—H5	121.9
C3—N3—C5	109.47 (15)	C4—C5—N3	116.16 (17)
S1—C1—S2	129.53 (9)	C4—C5—H5	121.9
N1—C1—S1	114.64 (13)	C2—C6—H6A	109.5
N1—C1—S2	115.66 (14)	C2—C6—H6B	109.5
N2—C2—S1	114.04 (14)	C2—C6—H6C	109.5
N2—C2—C6	123.05 (17)	H6A—C6—H6B	109.5
C6—C2—S1	122.91 (14)	H6A—C6—H6C	109.5
S3—C3—S2	117.96 (10)	H6B—C6—H6C	109.5
N3—C3—S2	126.86 (13)		
			
S3—C4—C5—N3	−1.7 (2)	C2—S1—C1—S2	−173.80 (14)
N1—N2—C2—S1	1.3 (3)	C2—S1—C1—N1	1.12 (17)
N1—N2—C2—C6	−179.1 (2)	C3—S2—C1—S1	−13.37 (15)
N2—N1—C1—S1	−0.7 (2)	C3—S2—C1—N1	171.75 (15)
N2—N1—C1—S2	174.98 (15)	C3—S3—C4—C5	1.02 (15)
C1—S1—C2—N2	−1.35 (17)	C3—N3—C5—C4	1.6 (3)
C1—S1—C2—C6	179.1 (2)	C4—S3—C3—S2	178.37 (11)
C1—S2—C3—S3	156.31 (10)	C4—S3—C3—N3	−0.16 (15)
C1—S2—C3—N3	−25.35 (18)	C5—N3—C3—S2	−179.10 (14)
C1—N1—N2—C2	−0.4 (3)	C5—N3—C3—S3	−0.7 (2)

### 5-Methyl-2-[(1,3-thiazol-2-yl)sulfanyl]-1,3,4-thiadiazole Hydrogen-bond geometry (Å, º)

**Table:**

*D*—H···*A*	*D*—H	H···*A*	*D*···*A*	*D*—H···*A*
C4—H4···N2^i^	0.93	2.55	3.472 (2)	169
C5—H5···N3^ii^	0.93	2.63	3.392 (3)	139

Symmetry codes: (i) *x*+1, *y*, *z*; (ii) −*x*+1, −*y*, −*z*+1.
